# Retraction Note: IFIT1 modulates the proliferation, migration and invasion of pancreatic cancer cells via Wnt/β-catenin signaling

**DOI:** 10.1007/s13402-023-00897-4

**Published:** 2023-10-30

**Authors:** Tian-Hao Li, Bang-Bo Zhao, Cheng Qin, Yuan-Yang Wang, Ze-Ru Li, Hong-Tao Cao, Xiao-Ying Yang, Xing-Tong Zhou, Wei-Bin Wang

**Affiliations:** 1 Department of General Surgery, State Key Laboratory of Complex Severe and Rare Diseases, Peking Union Medical College Hospital, Chinese Academy of Medical Science and Peking Union Medical College, Beijing, China; 2 Department of Breast Surgery, Peking Union Medical College Hospital, Chinese Academy of Medical Science and Peking Union Medical College, Beijing, China

**Retraction Note: Cellular Oncology (2021) 44:1425–1437**

10.1007/s13402-021-00651-8

The Editors-in-Chief have retracted this article at the authors’ request. After publication, the authors contacted the journal requesting a correction to Figs. 4 and 5 due to image overlap with their other article, specifically:

- Figure 4d 48 h IFIT1 image appears highly similar to Fig. 7f Huh7 48 h control in [1].
- Figure 5c Aspc-1 si-IFIT1 image appears highly similar to Fig. 7h Huh7 si-METTL18 in [1].
- Figure 5a and c Aspc-1 si-IFIT1 images appear to overlap.

Further checks by the Publisher found several additional cases of image overlap in Figs. 2, 4 and 5, as well as other similarities between Figs. 4 and 7 in this article and Fig. 7 in [1]. The authors have provided the underlying raw data for validation, but due to the high number of errors in the published figures, the authors have decided to retract this article.

The authors have been invited to resubmit a corrected version of the article for peer review.

All authors agree to this retraction.

## References

[1] Li T-H, Qin C, Zhao B-B, Cao H-T, Yang X-Y, Wang Y-Y, Li Z-R, Zhou X-T, Wang W-B (2021). Identification METTL18 as a potential prognosis Biomarker and Associated with Immune infiltrates in Hepatocellular Carcinoma. Front. Oncol..

